# Examination of social media usage habits of generation Z

**DOI:** 10.3389/fpsyg.2024.1370823

**Published:** 2024-07-15

**Authors:** Metin Elkatmış

**Affiliations:** Department of Primary Education, Faculty of Education, Kırıkkale University, Kırıkkale, Türkiye

**Keywords:** habit, primary school, student, social media, Generation Z

## Abstract

Undoubtedly, social media is one of the significant components of computer and internet technologies. It offers users the opportunity to share emotions, thoughts, and life experiences through various means such as text, images, and videos on the internet. In doing so, it not only provides a multifaceted communication and interaction opportunity for users of all ages but also creates a dynamic and entertaining parallel universe. This universe shapes and transforms individual, social, and cultural life, as well as habits. With the daily increase in user numbers, social media has become an integral part of our lives. The primary objective of this study is to determine the social media usage habits of fourth-grade students, known as Generation Z. The research was conducted using semi-structured interviews, a qualitative data collection method. The universe of the study consisted of 654 fourth-grade students attending state schools in seven different provincial centers representing the seven geographical regions of Turkey during the 2021–2022 academic year. The data were collected using an interview form developed for this research and analyzed with the content analysis technique. According to the research findings, it was determined that approximately three-quarters of primary school fourth-grade students use social media on a daily basis. Moreover, most participants stated that they use social media for educational, communicative, and entertainment purposes. The most preferred social media platforms by students were identified as YouTube, WhatsApp, TikTok, and Instagram.

## Introduction

One of the most significant inventions of the recent past is undoubtedly the internet. Although it was initially developed for military purposes in 1969, it has now penetrated every aspect of life and achieved widespread use. This has led to the emergence of a new, different, and parallel culture as traditional ways of life transitioned into digital environments. In this technology-centered new world, the structure, functioning, and content of social life have undergone radical changes. It would not be an exaggeration to say that the concept of communication is the one most affected by this transformation. For many years, human expression was carried out through language, writing, and the printing press, but nowadays, it takes place through the internet, computers, and communication technologies. Especially in recent years, the main hub of communication has shifted to social media or social networking platforms accessible through the internet. In this study, the social media usage habits of Generation Z have been focused upon, examining factors such as the duration of use on social media platforms, the frequency of daily visits, preferred platforms, objectives, content interaction, communication, and the relationship with followers.

In the literature, the new form of communication, referred to by various names such as social media, social network, and social sharing sites, will be used as “social media” in this study due to reasons such as its broad scope and widespread usage. This relatively new concept is defined in different ways in the literature. Social media is a modern concept where internet users can create and share their content, interact with other users, and communicate through various platforms (Boyd and Ellison, 2007; Kaplan and Haenlein, 2010; Kietzmann et al., 2011). According to Sayımer (2008), social media draws attention as a common term used for all kinds of online tools and websites that allow users to share emotions, thoughts, interests, and information, thereby communicating with each other. With a general approach, social media represents the newest and most vibrant dimension of post-modern culture, offering people the opportunity to share their feelings and thoughts through multiple mediums such as text, images, sound, and video, and is utilized by individuals of all ages.

Social media serves a multifaceted function, extending beyond providing users with the means to express themselves; it encompasses a broad spectrum from communication to entertainment, and from education to commerce. For instance, YouTube transcends its role as merely a site for entertainment-oriented video sharing, emerging as a platform that caters to educational content across all types and levels (Duffy, 2008). Users can create, edit, and publish their own content through social media; they can engage with other users, comment, like, and send messages; they can acquire knowledge on various topics, stay updated with current news, and receive education. Consequently, users can showcase their creativity, generate knowledge, build social networks, forge friendships, join communities, access information with ease, support their learning processes, and be exposed to diverse perspectives (Boyd and Ellison, 2007; Kaplan and Haenlein, 2010; Kietzmann et al., 2011). With these and many more features, social media constitutes one of the most potent tools of modern communication (Tosyalı and Sütçü, 2016).

As a reference to the proposition that every new technology brings its own habits (Özdenören, 2016), social media habit represents a new type of habit introduced by technology. In general, the concept of habit refers to a characteristic behavioral pattern acquired through continuous and regular repetition of something. Social media habit can be defined as being accustomed to relevant platforms and engaging in their continuous and regular use. Especially with the increasing ease of access to computer and internet technologies and the frequency of usage, social media habits have also been on the rise. To examine trends and usage habits in the digital world, the “Digital 2022: Global Overview” report prepared by DataReportal in collaboration with “We Are Social” and “Hootsuite” indicates that as of January 2022, 62.5% of the world population are internet users, and 58.4% are social media users (Kemp, 2022). These figures show a more than twofold increase in internet users and more than threefold increase in social media users compared to 2012.

### Media use in childhood

Technology plays a significant role not only in the lives of adults but also in the lives of children. Initially interacting with television and being entertained by it, children’s interests soon include computers, the internet, and mobile phones (Güler Küçükturan, 2018). Indeed, the Turkish Statistical Institute (TÜİK)'s “Information Technology Usage and Media in Children Aged 06–15, 2013“study (Türkiye İstatistik Kurumu (TÜİK), 2013) found that the average age of mobile phone usage among children aged 06–15 is seven, computer usage starts at age eight, and internet usage begins at age nine. According to the Child Trends Data Bank report prepared in 2015, while internet usage rates are steadily increasing across all age groups, the most significant increase has been observed in children’s usage. The report also noted that in some European countries, the initial age of internet use has dropped to eight (Child Trends DataBank, 2015; cited in Üstündağ, 2020). Similarly, two separate studies by the Pew Research Center in 2015 and 2022 indicate that daily internet usage among American teenagers aged 13–17 has risen from 92 to 97% (Pew Research Center, 2015, 2022). In a like manner, Türkiye İstatistik Kurumu (TÜİK) (2021) “Children in Statistics, 2021″ report shows that the internet usage rate among children aged 6–15 was 50.8% in 2013, which increased to 82.7% in 2021. These figures suggest that the rise in computer and internet technology usage will inevitably influence social media usage as well.

Indeed, numerous studies indicate that social media usage among children is prevalent in Turkey. Numerous studies indicate that social media usage among children is prevalent in Turkey. For instance, the research conducted by Kaşıkcı et al. (2014) illustrates that a significant portion of children become members of social media platforms before the age of 13. Similarly, Okumuş and Parlar (2018) reveal that 86% of children aged between 11 and 14 are members of social media sites, with a majority of unregistered individuals actively engaging on these platforms, some for more than 4 years. Özdemir (2021) emphasize children’s preference for accessing the internet, particularly through platforms such as Twitter, Facebook, and Instagram. Baştürk Akca et al. (2015) research demonstrates that children maintain multiple accounts on social networks, visiting them at least once daily to communicate with acquaintances. Research outcomes from various countries echo similar trends to those in Turkey. For example, You et al. (2023) argue that social media usage is dense and widespread among Dutch children. According to data from 2015, 71% of American youth were identified as active users of multiple social media platforms (Pew Research Center, 2015). Li (2007) also suggests that young people use social media more frequently than adults. A survey conducted by Common Sense Media with 1,030 children aged 13–17 revealed that 90% of the children were social media users, and 75% had their own social media accounts (Rideout, 2012). Another study by the same organization in 2020 found that children aged 5–8 were using social media platforms such as Instagram, Snapchat, and TikTok, albeit to a limited extent (Rideout and Robb, 2020). From this data, it can be said that social media holds a significant place not only in the lives of adults but also in the daily lives of children, and its use is increasing every day.

Hegel (2019) states that “everyone is a child of their own time,” highlighting how individuals are shaped by the society and conditions they live in. This thought, introduced by Hegel two centuries ago, is relevant to the frequently discussed concept of “generations” today. The terms “generation” or “cohort” are used to describe groups of people born in a particular period who share similar historical, social, and cultural experiences (Adıgüzel et al., 2014). The Turkish Language Association’s Contemporary Dictionary (Türk Dil Kurumu (TDK), 1998) lists “generation” alongside “progeny,” “offspring,” and “generation,” referring to a community of individuals born around the same time, sharing similar conditions. Moreover, this term usually encompasses groups of people spanning 25 to 30 years. In this context, the term can be considered a phenomenon used to categorize generations in relation to the times and events they experience. Although various classifications exist in different sources, the classification generally accepted was proposed by Oblinger and Oblinger (2005). According to this classification, those born between 1925–1946 are called the Silent Generation, 1947–1964 the Baby Boomers, 1965–1980 Generation X, 1981–1995 Generation Y, and those born after 1995 are referred to as Generation Z.

Each generation is distinguished by distinct characteristic features. For instance, Generation Z, the target audience of this study, is a cohort born into technology and heavily interacting with digital environments (Twenge, 2017). This generation, having grown up during a time when the internet and mobile devices were widely used, has developed digital skills from an early age. While actively using social media platforms more than other generations, members of Generation Z craft their digital identities in creative ways and construct their posts with original content (Seemiller and Grace, 2016). Research indicates that Generation Z has shorter attention spans compared to other generations (Twenge, 2017). Furthermore, individuals of Generation Z are noted to be more independent and entrepreneurial, open to innovations, and sensitive to global issues (Seemiller and Grace, 2016). Consequently, Generation Z stands as the youngest and newest actors representing the spirit and dynamism of the era they exist in.

The social media usage habits of a generation raised with computer and internet technologies emerge as one of the most significant phenomena of the digital age. Both the research results mentioned above and the integration of childhood with digital technology demonstrate that social networks have enveloped our lives. This established high level of connection brings with it cultural, social, economic, and psychological effects (Aydın, 2016). A healthy, balanced, and sustainable relationship is possible only if the extent of children’s social media habits is known. However, it is known that both general and academic research are conducted on groups aged 14–15 and above. Yet, the phenomenon of social media, which appeals to people of almost every age, needs to be examined in younger age groups as well. This study has emerged to describe the social media usage habits of fourth-grade elementary students, referred to as Generation Z. In this context, the research sought answers to the questions defined below:

1. How long have fourth-grade students been using social media applications?
2. How frequently do fourth-grade students check social media applications on a daily basis?
3. Which social media applications are most commonly preferred by fourth-grade students?
4. What are the purposes of social media usage among fourth-grade students?
5. How do fourth-grade students engage with content on social media platforms? Do they leave comments?
6. Whom do fourth-grade students communicate with through social media for messaging and in group chats?
7. Do fourth-grade students maintain communication with the people they follow on social media in their daily lives?

## Materials and methods

### Research design

This study was conducted using a qualitative data collection technique called semi-structured interview. Qualitative research is considered a fundamental approach to understanding life, as it aims to present perceptions and events in a natural and comprehensive manner within their realistic context. There are three main types of data collection methods commonly used in qualitative research: interviews, observations, and document analysis (Yıldırım and Şimşek, 1999; Merriam, 2013). The interview method itself can be further categorized into structured, semi-structured, and unstructured interviews. Semi-structured interviews are preferred by researchers because they allow for the reordering and further discussion of questions during the interview process (Ekiz, 2017). Moreover, interviews are chosen as a method because they offer the opportunity to deeply explore an individual’s knowledge, emotions, and attitudes. They allow researchers to gain in-depth insights into the participants’ perspectives.

### Study group

The study group in this research is a limited population, which is also referred to as the study universe in the literature (Arseven, 1993; Karasar, 1999). The study universe represents the accessible population, and research is conducted on this defined universe, with the results only being generalized to this limited population (Karasar, 1999). The study population for the current research comprises fourth-grade students attending schools in seven different provincial centers, each representing one of Turkey’s seven geographical regions during the 2021–2022 academic year. The age of the students comprising the study group ranges from 9 to 10 years old. Turkey has been divided into seven distinct geographical regions since the First Geography Congress in 1941, which was organized and accepted based on various factors such as geographical location, climate, vegetation, and human factors. These regions are named the Mediterranean, Black Sea, Aegean, Marmara, Central Anatolia, Eastern Anatolia, and Southeastern Anatolia (Özey, 2016). The selection of the study population to represent the extensive geographical structure of Turkey was planned in two stages. In the first stage, seven cities representing seven different geographical regions were identified, and the lists of primary schools in each city were obtained. Based on these lists, three primary schools were selected from each city. Since accessing student lists would not be easy and practical, fourth-grade classes in the selected schools were included in the sample. Subsequently, one class representing fourth graders from each school was randomly selected, and all students in the selected classes constituted the study population of the research. Accordingly, the demographic characteristics of the participants are provided in Table 1.

**Table 1:** Demographic information of the students constituting the study group.

Gender	n	%	Having a smartphone	n	%
Female	310	47.40	Yes	126	19.27
Male	344	52.60	No	528	80.73
**Total**	**654**	**100**	**Total**	**654**	**100**
**Place of Residence**	**n**	**%**	**Ownership of computer/tablet**	**n**	**%**
District	289	44.16	Yes	338	51.68
City	184	28.12	No	316	48.32
Metropolitian City	181	27.72	**Total**	**654**	**100**
**Total**	**654**	**100**			
**Family’s socio-economic level**	**n**	**%**	**Availability of internet at home**	**n**	**%**
Lower income group	157	24.00	Yes	560	85.63
Middle income group	463	70.80	No	94	14.37
Upper income group	34	5.20	**Total**	**654**	**100**
**Total**	**654**	**100**			

Upon examining the data in Table 1, it is understood that the gender distribution of the participants in the study is approximately equal, the settlement where they reside is largely district-level, and the socio-economic status of the family is mostly in the middle-income group. Regarding the use of technology, it has been determined that the participants’ ownership rates of smartphones and computers/tablets are low, whereas the availability of internet at home is high.

### Data collection

To collect research data, a semi-structured interview form was developed by the researcher. The interview form was specifically designed for fourth-grade elementary school students based on an extensive literature review (Ravalli and Paoloni, 2016; Güney, 2020; Muhametjanova et al., 2020; Üstündağ, 2020). The process of developing the form involved creating a question pool and then structuring it into a semi-structured interview form, following the relevant literature review. The form’s content validity was ensured by seeking opinions from eight expert academicians. Necessary adjustments were made based on the feedback received from the experts. Finally, to check the clarity of the questions, five pilot interviews were conducted to give the form its final shape. During the preparation of the interview questions by the researcher, attention was paid to the following characteristics: being relevant to the study’s purpose, clear, unbiased, open-ended, descriptive, and single-purpose questions (Karasar, 1999; Merriam, 2013). The survey form developed based on these criteria includes topics such as the duration of social media application usage, daily usage frequency, preferred applications, purpose of use, habits of commenting on content, and the communication status with individuals and groups, for fourth-grade elementary students. This meticulous approach ensures that the translated article will maintain the high level of precision required for academic publication.

### Data collection process

Interviews were conducted in the researcher’s office and through online video conferencing programs, which allowed participation from some attendees at school and others at home. Parents and teachers assisted in preparing the necessary technical and hardware tools and creating a suitable environment, aiming to prevent potential issues arising from the participants or the location. At the beginning of the application process, a brief and clear briefing was provided on the purpose of the interview, the data collection process, the confidentiality of the data, and the conditions for announcing the results of the study, ensuring that participants felt comfortable, secure, and free. The dates and times of the interviews were determined according to the participants’ preferences, with care taken to create an environment as genuine as possible. The researcher recorded the responses using both a voice recorder and note-taking techniques. This method is considered the most ideal in interview-type research (Yıldırım and Şimşek, 1999). In interviews lasting approximately 8 to 12 min, participants’ demographic information was obtained through brief questions. Moreover, during the interview, the researcher paid special attention to not being directive, keeping the topic from straying from its purpose, and ensuring equal speaking rights and time for all participants (Yıldırım and Şimşek, 2005; cited in Yılmaz and Altınkurt, 2011).

### Data analysis

In the evaluation of the data obtained, the content analysis method, which is frequently used in qualitative research, has been employed. Content analysis is a research technique that researchers use to identify recurring patterns, themes, and meanings within a specific text or dataset (Braun and Clarke, 2006). The primary goal of this method is to reach concepts and relationships that can explain the collected data. The process involves grouping similar data within certain concepts and themes and organizing them in a way that is comprehensible to the reader (Yıldırım and Şimşek, 1999). In this study, the data obtained were coded within certain concepts based on their frequency of occurrence and presented with frequency and percentage values.

### Trustworthiness and ethics

In scientific research, one of the most sought-after qualities is reliability. In this context, to ensure the reliability of data, records have been kept and the transcription of the record has been reviewed by another expert, and the two transcriptions have been compared. In qualitative research, the issue of reliability in terms of the credibility of the results holds a special meaning (Yıldırım and Şimşek, 1999). In this study, the formula by Miles and Huberman (1994), [Consensus / (Disagreement + Consensus) X 100], was used to ensure reliability. The reliability between the two transcriptions was calculated to be 84%. Since the consistency rate among coders should not be below 80% (Miles and Huberman, 1994), the findings of the research have been considered reliable. To ensure full compliance with ethical principles in the research, approval was obtained from the Kırıkkale University Social and Human Sciences Research Ethics Committee after the preparation of data collection tools.

### Findings

Below, the findings and comments reflecting the opinions of Generation Z regarding their social media usage habits have been analyzed in accordance with the predetermined sub-problems and presented, respectively. Accordingly, the data on the duration of participants’ use of social media applications is summarized in Table 2.

**Table 2:** The duration of the participants’ use of social media applications.

Time to use social media applications	n	%
I do not use yet	288	44.03
I’ve been using it for less than a year	82	12.53
I’ve been using it for a year	63	9.64
I’ve been using it for 2 years	98	14.98
I’ve been using it for 3 years	67	10.25
I’ve been using it for over 4 years	56	8.57
**Total**	**654**	**100**

According to Table 2, nearly half of the fourth-grade elementary students participating in the study (44.03%) have not yet used social media applications. This rate is significantly higher compared to other categories. However, looking at the overall total of those who stated they use social media applications, slightly more than half (55.97%) are using them. From this perspective, it can be said that social media usage is quite common among fourth-grade elementary students.

Another question of interest within the scope of the research is related to how often fourth-grade elementary school students check their social media applications daily. The results obtained regarding this matter are presented in Table 3.

**Table 3:** Daily social media usage frequency of fourth-grade elementary school students.

**Frequency of using social media applications**	**n**	**%**
I never look	175	26.8
Less than an hour	177	27.1
1–2 h	195	29.8
3–4 h	76	11.6
5 h or more	31	4.7
**Total**	**654**	**100**

When Table 3 is evaluated in terms of the total number of participants who indicate daily social media usage, it can be concluded that nearly three-quarters (73.24%) of the participants use social media regularly. Conversely, those who do not use social media daily represent a rather limited group, constituting one-quarter (26.8%) of all participants. These data reveal that a significant majority of the respondents use social media on a daily basis, yet the duration of usage varies among them.

The research also examined the most preferred social media applications among fourth-grade elementary school students, and the results obtained in this regard are presented in Table 4.

**Table 4:** Social media application preferences of fourth-grade elementary school students.

Most preferred social media applications	1st preference		2nd preference		3rd preference		Total	
	n	%	n	%	n	%	n	%
YouTube	353	53.98	106	16.20	41	6.27	**500**	**76.45**
WhatsApp	171	26.15	94	14.37	37	5.65	**302**	**46.17**
Tiktok	78	11.92	55	8.40	37	5.65	**170**	**25.99**
Instagram	63	9.63	62	9.48	32	4.89	**157**	**24.00**
Facebook	37	5.65	15	2.29	29	4.43	**81**	**12.38**
Snapchat	31	4.74	22	3.36	29	4.43	**82**	**12.53**
Pinterest	14	2.14	10	1.52	18	2.75	**42**	**6.42**
Telegram	13	1.98	7	1.07	12	1.83	**32**	**4.89**
Bip	12	1.83	7	1.07	15	2.29	**34**	**5.19**
Wikipedia	12	1.83	8	1.22	12	1.83	**32**	**4.89**
Twitter	8	1.22	9	1.37	15	2.29	**32**	**4.89**
Messenger	8	1.22	10	1.52	18	2.75	**36**	**5.50**
Skype	6	0.91	8	1.22	11	1.68	**25**	**3.82**

Table 4 reflects the social media preferences of fourth-grade elementary students. According to this, the most frequently used social media applications by the participants are YouTube (53.98%), WhatsApp (26.15%), TikTok (11.92%), and Instagram (9.63%) respectively. It has been determined that the least used social media applications by the students are Skype (0.91%), Messenger (1.22%), and Twitter (1.22%).

The opinions of fourth-grade elementary school students regarding the purpose of using social media have also been examined, and the findings are presented in Table 5.

**Table 5:** Purposes of social media usage among fourth-grade elementary school students.

Purpose of using social media	1st preference		2nd preference		3rd preference		Total	
	n	%	n	%	n	%	n	%
For information sharing (homework, projects, etc.)	233	35.62	91	13.91	50	7.64	**374**	**57.18**
To communicate with my friends	141	21.55	64	9.78	16	2.44	**221**	**33.79**
To play games	130	19.87	36	5.50	47	7.18	**213**	**32.56**
For the purpose of accessing information	76	11.62	50	7.64	50	7.64	**176**	**26.91**
In order to exchange ideas on the subjects I am interested in.	64	9.78	44	6.72	33	5.04	**141**	**21.55**
To make new friends	55	8.40	10	1.52	14	2.14	**79**	**12.07**
For video sharing purposes	44	6.72	26	3.97	15	2.29	**85**	**12.99**
For the purpose of sharing music	41	6.26	18	2.75	25	3.82	**84**	**12.84**
For photo sharing purposes	37	5.65	25	3.82	30	4.58	**92**	**14.06**
To find solutions to my daily problems	37	5.65	18	2.75	17	2.59	**72**	**11.01**
In order to contribute to my personal development	34	5.19	16	2.44	15	2.29	**65**	**9.93**
To follow the organizations I support	27	4.12	27	4.12	15	2.29	**69**	**10.55**
In order to determine my shopping preferences	26	3.97	16	2.44	17	2.59	**59**	**9.02**
To express myself	23	3.51	17	2.59	20	3.05	**60**	**9.17**
For instant status sharing	21	3.36	17	2.59	13	1.98	**51**	**7.79**
Other	40	6.11	9	1.37	14	2.14	**63**	**9.63**

According to Table 5, when examining the frequency of social media usage purposes among fourth-grade elementary students, it has been determined that participants primarily use social media for information sharing (homework, projects, etc.) with a percentage of 57.18%. The second most common use is for communication with friends, accounting for 33.79%, and the third is for playing games, at 32.56%. Additionally, it has been observed that participants use social media at the lowest rates for sharing instant status updates (7.79%), determining shopping preferences (9.02%), self-expression (9.17%), other purposes (9.63%), and personal development (9.93%).

Based on this data, it can be said that students prefer social media for academic, social, and entertainment purposes. Additionally, it is evident that social media is more commonly used for accessing information and exchanging ideas on subjects of interest compared to other purposes. Various reasons such as making new friends, sharing videos, music, and photos, finding solutions to everyday problems, and showing support are also observed as factors for their preference toward social media. These findings indicate that the purposes of social media usage among fourth-grade elementary school students are diverse.

The research also examined the situation of fourth-grade elementary school students in terms of commenting on content present on social media. The data obtained within this scope is presented in Table 6.

**Table 6:** Fourth-grade elementary school students’ status of commenting on content on social media.

Commenting on content on social media	n	%
Always	34	5.20
Sometimes	106	16.21
Rarely	104	15.90
Never	410	62.69
**Total**	**654**	**100**

As can be understood from Table 6, the majority of students (62.69%) indicate that they never comment on content found on social media. The total percentage of those who comment at varying levels is slightly more than a quarter (37.61%). These data suggest that the interaction levels of fourth-grade elementary students on social media vary and that the action of commenting is mostly not performed.

Furthermore, within the scope of the research, findings related to the individuals and groups with whom fourth-grade elementary school students communicate on social media were also analyzed using frequencies and percentages, and the results are presented in Table 7.

**Table 7:** Individuals and groups with whom fourth-grade elementary school students communicate on social media.

People and groups interviewed on social media	n	%
With my family	285	43.58
With my friend from school/neighborhood	227	34.71
With people from other cities and countries	87	13.30
With my new social network friends	55	8.41
**Total**	**654**	**100**

When examining Table 7, it is understood that the individuals and groups with whom fourth-grade primary school students communicate via social media are their families and close acquaintances. Conversely, the rate of communication with newly met social network friends and people from other cities and countries corresponds to approximately one-fifth of all participants.

The final question addressed in the research was whether fourth-grade elementary school students are also in communication with the individuals they follow on social media in their daily lives. The data regarding this matter are presented in Table 8.

**Table 8:** Status of fourth-grade elementary school students being in communication with individuals they follow on social media in daily life.

The state of being in contact with people followed on social media in daily life	n	%
I am in contact with all of them	129	19.72
I am in contact with some	161	24.62
I am in contact with very few of them	98	14.99
I’m not with any of them	266	40.67
**Total**	**654**	**100**

In Table 8, a majority of fourth-grade elementary students (40.67%) reported that they do not communicate in daily life with the individuals they follow on social media. Although the levels of communication vary, a general assessment reveals that more than half of the participants (59.33%) indicated they do communicate in daily life with the people they follow. Based on this data, it can be said that while a large majority of the participants are in communication with the people they follow in their daily lives, a significant group of students do not have any face-to-face interaction with them.

## Discussion, conclusion and suggestions

### Social media usage duration

This study, examining the social media usage habits of fourth-grade primary school students, has determined that participants use social media extensively. Indeed, research indicates that children use these platforms from an early age and frequently (Ulusoy and Bostancı, 2014; Dinleyici et al., 2016; Badri et al., 2017; Tuğlu, 2017; Treviño and Morton, 2019). For instance, data from the Turkish Statistical Institute (Türkiye İstatistik Kurumu (TÜİK), 2021) reveal that a majority of children aged 6–15 regularly use social media. Similarly, a trend was observed in Güney (2020) study with fifth-grade middle school students. It has been found that children in Turkey typically start using social media between the ages of seven and nine, and many children under the age of 13 have social media accounts (Ulusoy and Bostancı, 2014; Dinleyici et al., 2016). International studies support these findings; Treviño and Morton (2019) in Mexico, Livingstone et al. (2011) in Europe, and Ravalli and Paoloni (2016) in Argentina have obtained similar results. On the other hand, researchers find it interesting and noteworthy that the general age for social media account membership is usually 13, yet children younger than this are active users (Cagiltay et al., 2011; Ulusoy and Bostancı, 2014; Tuğlu, 2017; Treviño and Morton, 2019). In summary, these results indicate that the presence of children in the digital world and their social media usage habits begin at an early age and are widespread. This trend is not limited to the outcomes of this study alone but also aligns with the data from numerous national and international researches. Therefore, it can be posited that social media usage has become a global phenomenon of keen interest not only to adults but also to children. Consequently, it is of great importance for parents, educators, and policymakers to carefully examine this trend and to better understand and guide children’s digital lives.

### Daily social media usage frequency

Research has found that approximately three-quarters of fourth-grade elementary students use social media on a daily basis in a regular manner. This usage is generally limited to 1 or 2 h per day. In a study conducted with children aged 9–16 living in Turkey’s three major cities, Istanbul, Ankara, and Izmir, participants were observed to spend just over an hour on social media (Cagiltay et al., 2011). Güney (2020) study revealed that a significant portion of middle school students use social media for less than an hour a day, while another portion uses it for between one and 2 h. Similarly, a study in Canada found that the majority of middle and high school students spend less than 2 h per day on social media, but a quarter exceed this duration daily (Sampasa-Kanyinga and Lewis, 2015). A study by Türkiye İstatistik Kurumu (TÜİK) (2021) with Turkish children aged 6–15 showed that they use social media for an average of 2 h and 54 min on weekdays and 2 h and 44 min on weekends. Furthermore, a study on children in United Arab Emirates found that participants use social media for an average of 5.2 h per day, and this duration increases with age (Badri et al., 2017). To summarize, the duration of social media usage among students ranging from elementary to high school shows global similarities and occurs on a daily, intensive basis. This indicates that social media is an attractive activity for children and adolescents, becoming a significant part of their daily routines. However, it is crucial not to overlook the potential risks and possible addictive effects that excessive use of social media may bring. Furthermore, increased usage time may lead to a decrease in traditional face-to-face interactions and weaken social bonds between individuals, as well as reduce the time children allocate for more productive activities such as studying and reading. Therefore, it can be said that a careful examination of social media usage habits will assist children in maintaining a healthy and balanced life both online and offline, underscoring the need for further research in this area.

### Most used social media applications

The study has determined that the most frequently chosen social media applications among fourth-grade elementary students are YouTube, WhatsApp, TikTok, and Instagram. This outcome is consistent with the findings of research conducted with middle school students by Güney (2020) and Tuğlu (2017). Additionally, numerous international studies have shown that YouTube and Facebook are popular among children (Cagiltay et al., 2011; Livingstone et al., 2011; Ravalli and Paoloni, 2016; Treviño and Morton, 2019; Ayar et al., 2021). On the other hand, Badri et al. (2017) have determined that children in the United Arab Emirates prefer Instagram, YouTube, Snapchat, and Facebook more. These results suggest that children’s social media usage preferences exhibit similar trends regardless of geographic location. However, it is noteworthy that in this study, the TikTok application ranks higher compared to other research. Although this result contradicts the aforementioned research findings, it is in line with the findings of a study conducted with children in America by the Pew Research Center (2022), indicating that TikTok is one of the most popular networks among teenagers. Furthermore, all these data also parallel the most popular social media preferences worldwide (Kemp, 2023). To summarize, participants are increasingly preferring applications with visual and video content such as YouTube, TikTok, and Instagram, as well as communication and interaction-focused platforms like WhatsApp and Facebook. These preferences are consistent with research conducted in various countries. On the other hand, the rise of TikTok, a relatively new application compared to others, serves as an important indicator for understanding the changing trends and new media habits among the youth. Additionally, it is noteworthy that an information resource like Wikipedia is seldom chosen by school-aged children, which should be interpreted as a significant finding.

### Purposes of social media usage

One of the important findings obtained in the study is that fourth-grade elementary school students generally use social media platforms for educational content exchange, communication, and entertainment purposes, while to a lesser extent, they use them for instant status sharing, determining shopping preferences, self-expression, and personal development. This finding is consistent with the results of Tuğlu (2017) study with middle school students, which showed similarities in aiming for communication, accessing information, and sharing academic knowledge. In the same study, the use of social media for determining shopping preferences was found to have the lowest average. In a study conducted by Badri et al. (2017), participants stated that they primarily use social media to stay in touch with family and friends, seek information, and support learning. In the study by Cagiltay et al. (2011), it was observed that the majority of participants use social media to stay in touch with friends, with very few using it for playing games, education, or communicating with relatives. Based on these data, it can be said that social media is used as a multifaceted tool among students. It is understood that its use for fundamental needs such as acquiring and sharing information, communication, and entertainment is quite widespread. However, the fact that participants use it less for personal development, self-expression, and sharing instant statuses suggests that they use social media in a consumption-focused, passive manner. In this context, it is extremely important for educators and parents to guide and support children’s use of social media. Explaining the potential of social media to them and guiding them on its effective use can enable children to use these platforms more efficiently and safely. Thus, children will be raised as more equipped and conscious individuals in the digital world, and it will be possible for them to use social media not only as a tool for entertainment and communication but also as an environment for personal development and learning.

### Commenting behavior on social media

In the study, it was found that students’ engagement with content on social media varies, with the majority being passive users who do not leave comments. However, it was also observed that slightly more than a third of the participants tend to leave comments. The findings suggest that the research conducted by Bayzan et al. (2023) on 10,475 participants aged 8–16 supports the observation that children’s content creation and sharing on the internet are quite limited. Similarly, Güney (2020) indicates that more than half of the children did not share any content over a week-long period. Karabay (2019) attributes the predominant consumer tendency among children to a lack of sufficient skills in using digital technologies. On the other hand, Bayzan et al. (2023) also mention that young people do not allocate time for content production. While liking and commenting on posts are fundamental features of social media, the participants’ reluctance to reflect their personal thoughts on shared content does not align with the vibrant, dynamic, and participatory nature of social media (Gündüz and Pembecioğlu, 2013). Furthermore, it is noted that children’s use of social media primarily involves posting photos and videos and liking them (Nesi et al., 2018). Kuçlu (2016) criticizes the reduction of emotional responses to buttons and symbols on social media, suggesting that this passivizes individuals. This visual and symbol-heavy communication style shapes user behavior and promotes a culture dominated by visual communication. Additionally, the lack of direct studies on children’s likes and messages on social media is a significant issue that needs attention. Every action performed on the internet can be tracked as a digital footprint, which could lead to potential risks such as cyberbullying and privacy concerns for children’s personal information. Indeed, it is argued that among the risks associated with the misuse of the internet, cyberbullying (Can, 2022) and social media addiction (Taylan et al., 2017; Goodwin, 2021) stand out. Research indicates that intensive use of social media increases the likelihood of both perpetrating and being exposed to cyberbullying (Altundağ, 2016; Taylan et al., 2017). Similarly, Kırık et al. (2015) have found that as the time spent on the internet and social networking platforms increases, so does social media addiction. Furthermore, Üstündağ (2020) has identified a significant and positive relationship between technology usage and social media addiction. Therefore, it should be remembered that technological tools such as computers, the internet, and social media not only provide opportunities but also entail serious risks. Particularly, emphasis should be placed on education and awareness campaigns for children to promote correct, effective, and mindful use of social media. In this way, efforts can be made to contribute to children’s safe and balanced existence in the digital world.

### Individuals communicated with via social media

In the study, it was found that fourth-grade elementary school students primarily communicate through social media with close circles such as family, schoolmates, and neighborhood friends. This finding is also parallel to some research results in the literature (Cagiltay et al., 2011; Badri et al., 2017; Üstündağ, 2020). Baştürk Akca et al. (2015) found in their research that children do not communicate with strangers and reject friend requests from unfamiliar individuals due to not knowing them. On the other hand, it has been determined that the rate of meeting with new acquaintances from other cities and countries through social media is quite low. This suggests that children’s modes of establishing relationships on social media reflect their real-world relationships. In other words, children predominantly communicate with close circles such as family, schoolmates, and neighborhood friends on social media. This outcome can be interpreted from two different perspectives. Firstly, the tendency of children to communicate with their immediate environment reflects a significant situation as it can aid in the development of their social skills and the formation of strong social bonds. Kuss and Griffiths (2017) also state that the primary purpose of using social media is to maintain social connections, primarily with family and friends. Secondly, children’s avoidance of communication with strangers can be seen as a reflection of online safety awareness. Indeed, research shows that children often resolve issues encountered on social media by communicating with their parents and close friends (Üstündağ, 2020; Turgut and Kurşun, 2021). Karahisar (2014) in his study mentions that only a few children accept friend requests from strangers. Although this may seem like good news, it is known that there are many negative examples in the digital world that children experience. Therefore, all activities of children on the internet should be monitored, and necessary safety measures should be taken. For this purpose, primarily parents and educators should provide the necessary guidance to children on the use of social media. Moreover, creating a safe communication environment where children can openly discuss and seek support for the problems they encounter on social media platforms is of great importance.

### Communication status with social media followers

The final finding obtained in the study indicates that fourth-grade elementary school students are largely in communication with the individuals they follow on social media in their daily lives. Similarly, Ravalli and Paoloni (2016) found that the majority of Argentinean adolescents aged 13–17 only establish friendships with people they know. However, it was also found that a significant group of students had no face-to-face communication with the individuals they follow. This finding parallels the research result of Livingstone et al. (2011), which showed that European users aged 9–12 establish communication via social media with individuals with whom they have limited or no offline connections. In summary, children are primarily engaged in communication and interaction with familiar social circles, while they are also able to establish relationships beyond this circle, albeit to a limited extent. While social media forms a significant platform for children and adolescents to interact with their social circles, it is crucial to be vigilant about the content and safety of these interactions. Particularly, forming friendships with strangers is seen as one of the significant dangers awaiting children in social media environments (Akçay, 2020). Therefore, ensuring the online safety of children and teaching them to use the internet responsibly has become a fundamental duty incumbent upon adults.

In light of these findings, it is essential for parents, educators, and policymakers to carefully monitor children’s use of social media. Understanding the potential risks and opportunities of children’s social media use and providing appropriate guidance and support is crucial. Parents should monitor and set boundaries for their children’s social media use (Güler Küçükturan, 2018; Goodwin, 2021; Yılmaz, 2023). They should understand the purposes for which children use social media and provide necessary support in this regard. Additionally, they can use various control and filtering tools to supervise children’s online interactions and ensure their safety. Educators may consider integrating lesson materials and learning tools into social media platforms to encourage children to use social media for educational purposes. Policymakers can provide support for educational institutions and families in creating guidance and policies regarding social media use. They can also establish legal regulations and standards to ensure children’s online safety. In this way, children can be helped to use social media more consciously and safely, and the effective use of these platforms for education and communication can be promoted.

## Data availability statement

The raw data supporting the conclusions of this article will be made available by the authors, without undue reservation.

## Ethics statement

This study, which includes human participants, was reviewed and approved by the Kırıkkale University Ethics Committee on 22.04.2022, with decision number 04. The studies were conducted in accordance with the local legislation and institutional requirements. Written informed consent for participation in this study was provided by the participants’ legal guardians/next of kin. Written informed consent was obtained from the minor(s)’ legal guardian/next of kin for the publication of any potentially identifiable images or data included in this article.

## Author contributions

ME: Writing – original draft, Writing – review & editing.
